# FDA-approved drug repurposing screen identifies inhibitors of SARS-CoV-2 pseudovirus entry 

**DOI:** 10.3389/fphar.2025.1537912

**Published:** 2025-03-17

**Authors:** Manisha Singh, Shruthi Shanmukha, Raghda E. Eldesouki, Maged M. Harraz

**Affiliations:** ^1^ Department of Psychiatry, University of Maryland School of Medicine, Baltimore, MD, United States; ^2^ Department of Psychiatry, Johns Hopkins University School of Medicine, Baltimore, MD, United States; ^3^ Genetics Unit, Histology Department, Faculty of Medicine, Suez Canal University, Ismailia, Egypt; ^4^ Department of Pharmacology and Physiology, University of Maryland School of Medicine, Baltimore, MD, United States

**Keywords:** SARS-CoV-2, COVID-19, drug repurposing, FDA approved drug library, high-throughput screening, viral entry, ACE2 (angiotensin converting enzyme 2), tmprss2

## Abstract

**Background and purpose:**

The coronavirus disease 2019 (COVID-19) pandemic has devastated global health and the economy, underscoring the urgent need for extensive research into the mechanisms of severe acute respiratory syndrome coronavirus 2 (SARS-CoV-2) viral entry and the development of effective therapeutic interventions.

**Experimental approach:**

We established a cell line expressing human angiotensin-converting enzyme 2 (ACE2). We used it as a model of pseudotyped viral entry using murine leukemia virus (MLV) expressing SARS-CoV-2 spike (S) protein on its surface and firefly luciferase as a reporter. We screened an U.S. Food and Drug Administration (FDA)-approved compound library for inhibiting ACE2-dependent SARS-CoV-2 pseudotyped viral entry and identified several drug-repurposing candidates.

**Key results:**

We identified 18 drugs and drug candidates, including 14 previously reported inhibitors of viral entry and four novel candidates. Pyridoxal 5′-phosphate, Dovitinib, Adefovir dipivoxil, and Biapenem potently inhibit ACE2-dependent viral entry with inhibitory concentration 50% (IC_50_) values of 57nM, 74 nM, 130 nM, and 183 nM, respectively.

**Conclusion and implications:**

We identified four novel FDA-approved candidate drugs for anti-SARS-CoV-2 combination therapy. Our findings contribute to the growing body of evidence supporting drug repurposing as a viable strategy for rapidly developing COVID-19 treatments.

## Introduction

The coronavirus disease 2019 (COVID-19) pandemic, caused by the severe acute respiratory syndrome coronavirus 2 (SARS-CoV-2), has had a catastrophic impact on global health and the economy. Since its emergence in December 2019, the virus has spread rapidly, resulting in over 777 million confirmed cases and more than 7 million deaths worldwide as of January 2025 (World Health Organization, 2025). The ongoing pandemic has prompted extensive research into the interactions between SARS-CoV-2 and host cells, primarily focusing on understanding the mechanisms of viral entry and identifying potential therapeutic interventions (Jackson et al., 2022; Shang et al., 2020). The initiation of the viral life cycle hinges on the effective entry of SARS-CoV-2 into target cells, which begins with the binding of the virus to the cell surface receptor followed by endocytosis or direct fusion with the cell surface plasma membrane. Angiotensin-converting enzyme 2 (ACE2) binds the Spike (S protein), thereby acting as a receptor for SARS-CoV and SARS-CoV-2 (Hoffmann et al., 2020; Li et al., 2003). This interaction enables the virus to enter the cell and replicate, leading to infection and the characteristic symptoms of COVID-19. Moreover, proteolytic cleavage of the S protein on the cell surface by the transmembrane serine protease 2 (TMPRSS2) facilitates viral fusion to the membrane (Matsuyama et al., 2020). Understanding the molecular mechanisms of viral entry is crucial for developing targeted interventions to prevent or treat SARS-CoV-2 infection. The urgent need for effective treatments for COVID-19 has led to a rapid exploration of various strategies for therapeutic interventions against SARS-CoV-2. One primary focus has been inhibiting viral entry through targeting the interaction between the S protein and the ACE2 receptor. This approach has been explored using small molecule inhibitors, targeted antibodies, and emerging antiviral technologies (Zhang et al., 2021).

Drug repurposing, which involves using existing U.S. Food and Drug Administration (FDA)-approved drugs for new indications, has been evaluated as a potential strategy for treating COVID-19 (Gordon et al., 2020). High Throughput Screening (HTS) has played a significant role in drug discovery, particularly in identifying potential treatments for SARS-CoV-2 infection. HTS involves rapidly testing thousands of compounds to identify those with antiviral properties, which can potentially be repurposed for COVID-19 treatment (Riva et al., 2020). This approach allows for screening many compounds in a relatively short time, significantly expediting the drug discovery process. The historical FDA approvals for drug repurposing in treating COVID-19 reflect the rapid response to a global health crisis (FDA, 2021). These approvals were based on various clinical trials and emergency use authorizations (EUAs). Among the FDA-approved drugs for COVID-19, remdesivir is effective in reducing hospitalization rates and shortening recovery time (Beigel et al., 2020). Similarly, monoclonal antibodies such as bamlanivimab and casirivimab/imdevimab have also received EUAs to treat mild to moderate COVID-19 cases (FDA, 2020).

In this study, we screened the Johns Hopkins ChemCORE FDA-approved compound library containing 2,500 compounds to investigate their potential in inhibiting ACE2-dependent pseudotyped SARS-CoV-2 viral entry in HEK-293 cells. We identified several candidate compounds, including four FDA-approved drugs not previously known to inhibit ACE2-dependent pseudotyped SARS-CoV-2 entry. These FDA-approved compounds could serve as drug repurposing candidates for COVID-19 therapy.

## Methods

Murine leukemia virus (MLV) pseudovirus production in human embryonic kidney 293T (HEK-293T) cells: Cells at ∼60% confluence were transfected by the calcium-phosphate method with 50 μg of total DNA per 150 mm plate at a ratio of 3:2:1 by mass of a plasmid expressing MLV gag and pol proteins (pGag-Pol), the retroviral vector pQCXIX encoding firefly luciferase (pQC-Fluc), and a plasmid expressing the 2S-F-mutant protein of SARS-CoV-2 (pCAGGS SARS-2S-FM). Transfected cells were washed 18 h post-transfection and replenished with 20 mL DMEM supplemented with 10% fetal bovine serum (FBS) 2 mM L-glutamine, and penicillin/streptomycin. PV-containing culture supernatants were collected 48 h after changing media, cleared through 0.45 μm low protein binding filters, and used immediately. The plasmids pGag-Pol, pQC-Fluc, and pCAGGS-SARS-2S-FM are a kind gift from Dr. Michael Farzan, Boston Children’s Hospital, Harvard Medical School.

### Generation of stable cell lines

Stable cell lines for the overexpression of ACE2 and TMPRSS2 were generated using HEK-293 cells. Briefly, HEK-293 cells were transfected with *pCEP4-myc-ACE2 and pCEP4-TMPRSS2 (pCEP4-myc-ACE2 was a gift from Erik Procko (Addgene plasmid # 141185)* at a concentration of about 60% using polyfect reagent. The media was changed 18 h post-transfection with selection media, DMEM-low glucose, 10% FBS, 2 mM L-glutamine, and penicillin/streptomycin. The cells were initially selected with 1 mg/mL hygromycin (Corning #30–240-CR) and then maintained with 0.25 mg/mL hygromycin. Hygromycin selection was removed when cells were plated for pseudotyped viral entry experiments.

### High Throughput Screening (HTS)

We screened the Johns Hopkins ChemCORE compound library containing 2500 FDA-approved compounds for their effect on ACE-2-dependent pseudotyped SARS-CoV-2 viral entry as primary criteria. 20,000 cells were plated in each well of a 96-well plate and incubated overnight at 37°C 5% CO_2_. The cells were treated with 10uM of all the FDA drugs for 90 min at 37°C 5% CO_2_, followed by treatment with the chilled MLV pseudotyped SARS-CoV-2 virus encoding a luciferase reporter and 10uM drugs together. This process was performed on ice. The 96-well plates containing the compounds and virus were centrifuged at 3,000 rpm for 30 min at 4°C, followed by incubation at 37°C 5% CO_2_ for 2 h. Then, the media containing the virus and compounds was removed and replenished with fresh media, the cells were incubated further for 48 h at 37°C 5% CO_2_, followed by the end point analysis using the firefly luciferase assay. The results were normalized to the protein content in each well to account for any variability in the number of cells between different wells.

### Luciferase Assay

The luciferase assay was performed following the manufacturer’s recommendations. Briefly, at the endpoint of the experiment, the media was removed, and the cells were washed with PBS. Cells were then lysed using 1X passive lysis buffer (PLB) by rocking the plates for 20 min at room temperature. The cell lysate was transferred to a white opaque 96-well plate and added supplemented with the luciferase reagent (LAR-II solution) at a ratio of 1:4. After hand-rocking it for 1 min, the plate was read on a plate reader (Molecular Devices, SpectraMaxi3x, CA, United States). The plate was read at all luciferase wavelengths from the top of the plate for 1000 ms per well.

### Immunocytochemistry (ICC)

The cells were fixed using 4% PFA in 1x phosphate-buffered saline (PBS) pH 7.4 for 20 min at room temperature (RT), followed by washing with PBS. The fixed cells were incubated overnight at 4°C with a primary monoclonal antibody against myc-tag (9E10 clone, Invitrogen, United States). After the incubation period, ACE2 expressing 293T cells were washed three times with PBS and incubated with AF594 conjugated secondary antibodies (1:500, Abcam, United States) for 1 h at RT. Finally, after washing three times with PBS, cells were counterstained with 4′,6-diamidino-2- phenylindole (DAPI) (#10236276001, Sigma, United States) to visualize the cell nuclei. Cells were washed 3x with PBS. Stained cells were examined using a confocal fluorescence microscope (Zeiss LSM700).

### Immunoblotting

Cells were lysed on ice for 20 min in lysis buffer containing 50 mM Tris-HCl pH 8.0, 150 mM NaCl, 1% Triton X-100, protease inhibitors (complete™, EDTA-free Protease Inhibitor Cocktail from Sigma) and protein phosphatase inhibitor cocktail. The lysates were cleared by centrifuging at 16,000 x g for 15 min, followed by supernatant recovery. Protein Assay Dye Reagent (Bio-Rad) was used to normalize the total protein content. Protein samples were prepared for gel-loading by adding 1X final concentration of NuPAGE LDS Sample Buffer (Invitrogen) supplemented with β-mercaptoethanol (BME), followed by incubation at 95°C for 5 min. Thereafter, protein samples were resolved on a mini NuPAGE 4%–12% Bis-Tris gel (Thermo Fisher, Scientific United States) in the presence of 1X NuPAGE MES SDS running buffer (Thermo Fisher, Scientific United States). Proteins were then transferred to Immobilon-FL (Millipore). Membranes were blocked for 1 h at room temperature with 10% skimmed milk in TBS, followed by incubation with the primary antibodies for ACE2 (dilution of 1:1,000) and β-actin (dilution of 1:2000). Horseradish peroxidase (HRP)-conjugated secondary antibodies were used for detection with SuperSignal West Pico chemiluminescence reagent (Thermo Fisher, Scientific United States).

### Dose-Response and Toxicity Assays

Biapenem, Pyridoxal 5′-phosphate, Adefovir dipivoxil, and Dovitinib were used in dose-response and toxicity assays.

### Dose-Response

Different concentrations of the drugs were studied for the inhibition of pseudotyped SARS-CoV-2 viral entry. Briefly, 30,000 cells were plated in each well of 48-well plate and incubated overnight at 37°C 5% CO_2_. The cells were treated with 10pM-300 μM of all the FDA drugs for 90 min at 37°C 5% CO_2_, followed by treatment with the chilled SARS-CoV-2 pseudotyped MLV virus encoding a luciferase reporter and their respective 10pM-300 μM drugs together. This process was performed on ice. The 96-well plates containing the compounds and virus were centrifuged at 3,000 rpm for 30 min at 4°C, followed by incubation at 37°C 5% CO_2_ for 2 h. Then the media containing the virus and compounds was removed and replenished with fresh media; the cells were incubated further for 48 h at 37°C 5% CO_2_, followed by the endpoint analysis using the firefly luciferase assay. The results were normalized to the protein content in each well to account for any variability in the number of cells between different wells.

### Toxicity Assay

To test the toxicity, we plated 20,000 cells per well of a 96-well plate and incubated overnight at 37°C 5% CO_2._ Then, we treated the cells with drugs at 10nM-300 μM and incubated at 37°C 5% CO_2_ for 48 h. The toxicity assay was done using a cell titer glo cell viability kit from Promega per the manufacturer’s instructions.

### Statistical Analysis

Statistical analyses were performed in GraphPad Prism 10.2.3. Unpaired two-tailed Student’s t-test was used to compare two groups. One-way or two-way ANOVA with Bonferroni’s *post hoc* tests were used to compare more than two groups. The viral entry experiments were performed in two sets, first where the drug concentration ranged from 3nM to 300 μM (n = 4) and the second set from 3pM to 1 μM (n = 4). After normalizing to the vehicle treatment within each experiment, these sets were combined for the final dose response for each drug. Non-linear regression analysis was performed to determine the inhibitory concentration 50% (IC_50_), IC_90_, and concentration of cytotoxicity 50% (CC_50_) values for Biapenem, Pyridoxal 5′-phosphate, Adefovir dipivoxil, and Dovitinib.

## Results

### ACE2-dependent SARS-CoV-2 spike protein pseudotyped viral entry system

Pseudoviral *in vitro* models are well-established surrogates for investigating native viral entry mechanisms into host cells. These models are based on engineering replication-deficient chimeric viral particles with the genetic materials of one virus while bearing the surface protein(s) of a different virus and encoding a quantifiable reporter. The primary benefit of the pseudoviral *in vitro* models is that they enable the experiments to be performed using biosafety level 2 (BSL-2) protocols and facilities instead of the BSL-3 required to handle highly pathogenic viruses such as SARS-CoV-2. Since they only express the surface protein(s) of the native virus, the pseudoviral particles behave like their native counterparts for the host cell entry steps (Sedgwick et al., 2024).

**FIGURE 1:**
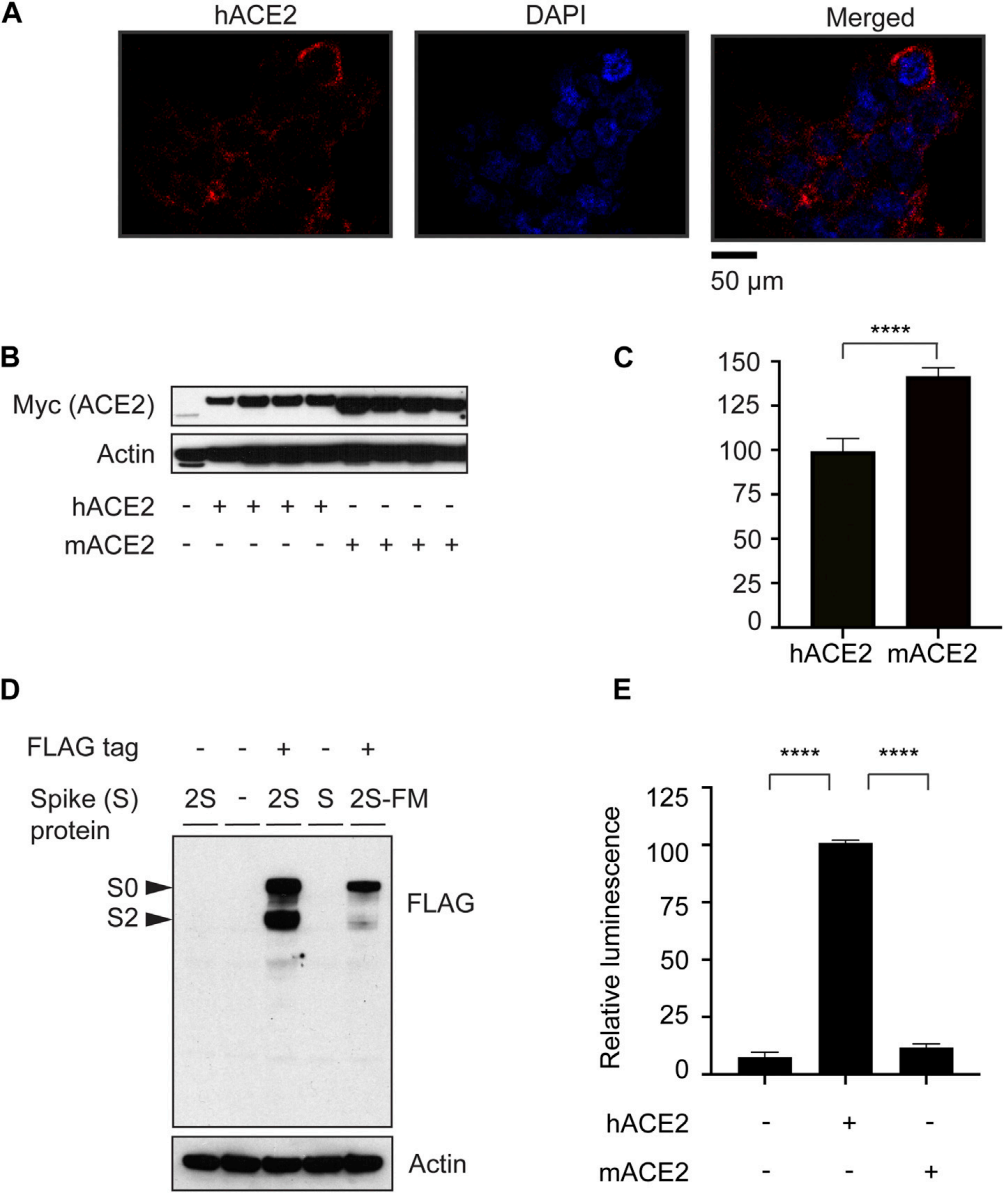
ACE2-dependent SARS-CoV-2 Spike protein pseudotyped viral entry system. **(A)** Immunostaining of HEK 293 cells overexpressing human myc-ACE2 using anti-myc antibody showing the plasma membrane distribution of the overexpressed ACE2 protein. **(B)** Validation of the expression of myc-tagged human and mouse ACE2 (hACE2 and mACE2, respectively) by western blotting. **(C)** Quantification of myc-tagged human and mouse ACE2. *n* = 4, Unpaired t-test, *P* < 0.0001, Error bars = +/−SEM. **(D)** Confirmation of FLAG-tagged-SARS-CoV-2 (2S) spike protein expression in 293 cells. 2S-FM is a furin protease mutant that is not cleaved into S0 or S2. **(E)** HEK-293 cells stably expressing hACE2 were transduced with MLV-encoding luciferase enzyme and pseudotyped with the SARS-CoV-2S-FM spike protein. *n* = 3, one-way ANOVA, *P* < 0.0001, Bonferroni’s multiple comparisons test, **** (*P* < 0.0001). Error bars = +/−SEM.

To study ACE2-mediated pseudotyped SARS-CoV-2 entry into cells, we established a HEK-293 cell line stably expressing myc-tagged human ACE2 (hACE2) (Mou et al., 2021). We confirmed the expression of hACE2 on the cell surface by immunocytochemistry (Figure 1A). Also, we characterized hACE2 vs. mouse ACE2 (mACE2) expression as shown by western blot analysis (Figures 1B,C). We verified FLAG-tagged SARS-CoV-2 Spike (S) protein expression in 293-cells by western blot (Figure 1D). As predicted, SARS-CoV-2S is cleaved into S0 and S2 bands. A furin-cleavage-site-mutant (SARS-CoV-2S-FM) showed one band (Figure 1D). Previous studies demonstrate that SARS-CoV-2S-FM is not critical for the efficiency of viral entry (Xia et al., 2020).

To monitor ACE2-mediated viral entry in HEK-293 cells stably expressing hACE2 or mACE2, we used MLV encoding a luciferase reporter, pseudotyped with SARS-CoV-2S-FM. Pseudotyped SARS-CoV-2 entry into cells is facilitated by human, but not mouse ACE2 (Figure 1E), consistent with previous reports showing that SARS-CoV-2 does not enter mouse cells (Rawle et al., 2021). These results show that we established a human ACE2-dependent pseudotyped SARS-CoV-2 entry model in HEK-293 cells.

### Screening of an FDA-approved drug library for inhibition of ACE2-dependent pseudotyped SARS-CoV-2 entry

**FIGURE 2:**
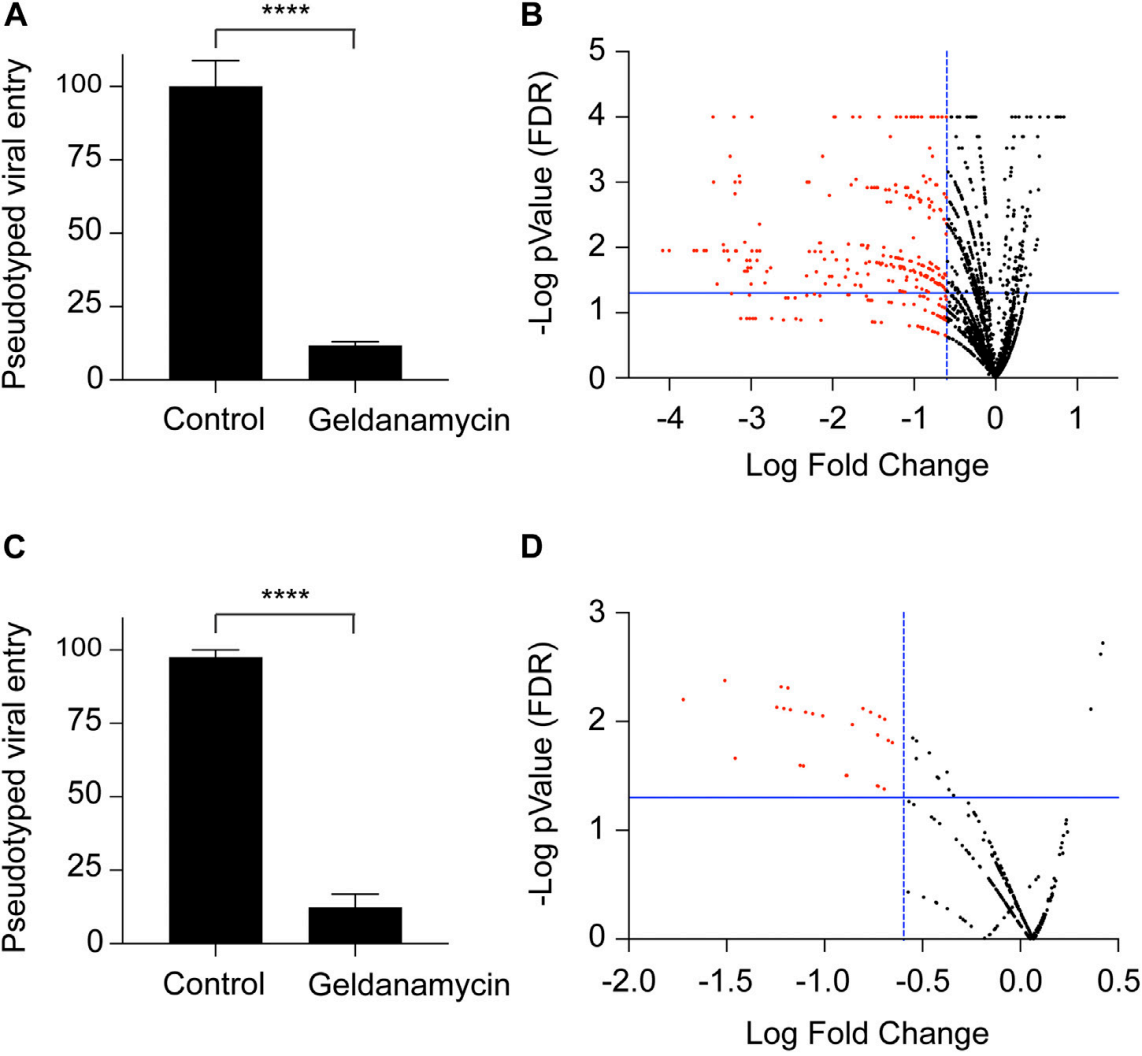
Screening of an FDA-approved drug library for inhibition of ACE2-dependent pseudotyped SARS-CoV-2 entry. **(A)** Graph confirming that the HEK-293-ACE2 cell system for the virus entry and inhibition of the viral entry in the presence of geldanamycin (10 μM), used as a positive control for the viral entry inhibition experiments. *n* = 27, **** (*P* < 0.0001), two-tailed t-test. Error bars = ±SEM. **(B)** Volcano plot for the screening of 2,500 drug library. The drugs were used at a concentration of 10 μM. Luciferase assay readings were normalized with their respective protein content. **(C)** Graph confirming that the HEK-293-ACE2 cell system for the virus entry and inhibition of the viral entry in the presence of geldanamycin (1 μM), used as a positive control for the viral entry inhibition experiments. *n* = 3, **** (*P* < 0.0001), two-tailed t-test. Error bars = ±SEM. **(D)** Volcano plot for the screening of 240 selected drugs from the library. The drugs were used at a concentration of 1 μM. Luciferase assay readings were normalized with their respective protein content.

We used the SARS-CoV-2S-FM pseudotyped viral entry system to screen a 2500 FDA-approved drug library for ACE2-dependent viral entry inhibitors. We used the NIH Molecular Libraries Probe Centers Network (MLPCN) Johns Hopkins ChemCORE FDA-approved drug library of nearly 2,500 compounds. We performed the initial screen twice in 96-well plates at a final concentration of 10μM, including negative and positive control wells in each plate. We used the firefly luciferase assay normalized to total protein concentration as a readout for the ACE2-dependent pseudotyped viral entry. Previous evidence suggests that heat shock protein 90 (Hsp90) inhibitors could serve as candidate therapeutics against human coronavirus infections (Li et al., 2020; Silva et al., 2022; Lubkowska et al., 2021; Alreshidi et al., 2021). We tested whether the HSP90 inhibitor geldanamycin inhibits ACE2-dependent pseudoviral entry. Our results show a 10-fold decrease in ACE2-dependent pseudotyped viral entry by geldanamycin (Figure 2A). We used geldanamycin as a positive control in our screen. We optimized the assay concerning cell number, pseudovirus concentration, and pre-incubation duration with the library compounds before pseudovirus addition and post-transduction. We obtained the best signal-to-noise ratio using 3 × 10^4^ cells/well in 250 μL of pseudovirus-containing media. We used the FDA-approved compounds at 10 μM final concentration. In the initial 96-well plates screen, we selected compounds that statistically significantly inhibited ACE2-dependent pseudotyped viral entry by at least four folds. Applying these criteria, 250 FDA-approved drugs were identified as potential hits in the initial SARS-CoV-2 pseudotyped viral entry assay (Figure 2B).

To narrow down the potential viral entry inhibitors to potent candidates, we repeated the test for the 250 potential hits selected from the 96-well plate screens (10 μM final concentration). We performed the assay in 24-well plates with a final compound concentration of 1 μM. We used geldanamycin at 1 μM final concentration as a positive control in each plate. Our findings demonstrate a 10-fold decrease in ACE2-dependent viral entry by 1 μM geldanamycin (Figure 2C). Like the initial screen, we selected compounds statistically significantly inhibited ACE2-dependent pseudotyped viral entry by at least four folds. We identified 18 FDA-approved drugs which that potently inhibit ACE2-dependent pseudotyped SARS-CoV-2 virus entry (Figure 2D).

### The effect of TMPRSS2 on the antiviral activities of the screen hits

TMPRSS2 proteolytically activate membrane fusion of SARS-CoV-2, facilitating viral entry (Hoffmann et al., 2020). To assess the effect of TMPRSS2 on the antiviral activities of the screen hits, we established a 293-cell line stably expressing human ACE2 and TMPRSS2. We tested the pseudotyped SARS-CoV-2 entry into these cells in the presence or absence of the TMPRSS2 activity inhibitor nafamostat mesylate or nafamostat at 1 μM concentration. Nafamostat is a comprehensive synthetic serine protease inhibitor, used widely as an anti-coagulant and for non-infectious indications like pancreatitis. Many groups have confirmed its role and potency in inhibiting SARS-CoV-2 (Mahoney et al., 2021; Wang et al., 2023; Yamamoto et al., 2020).

**FIGURE 3:**
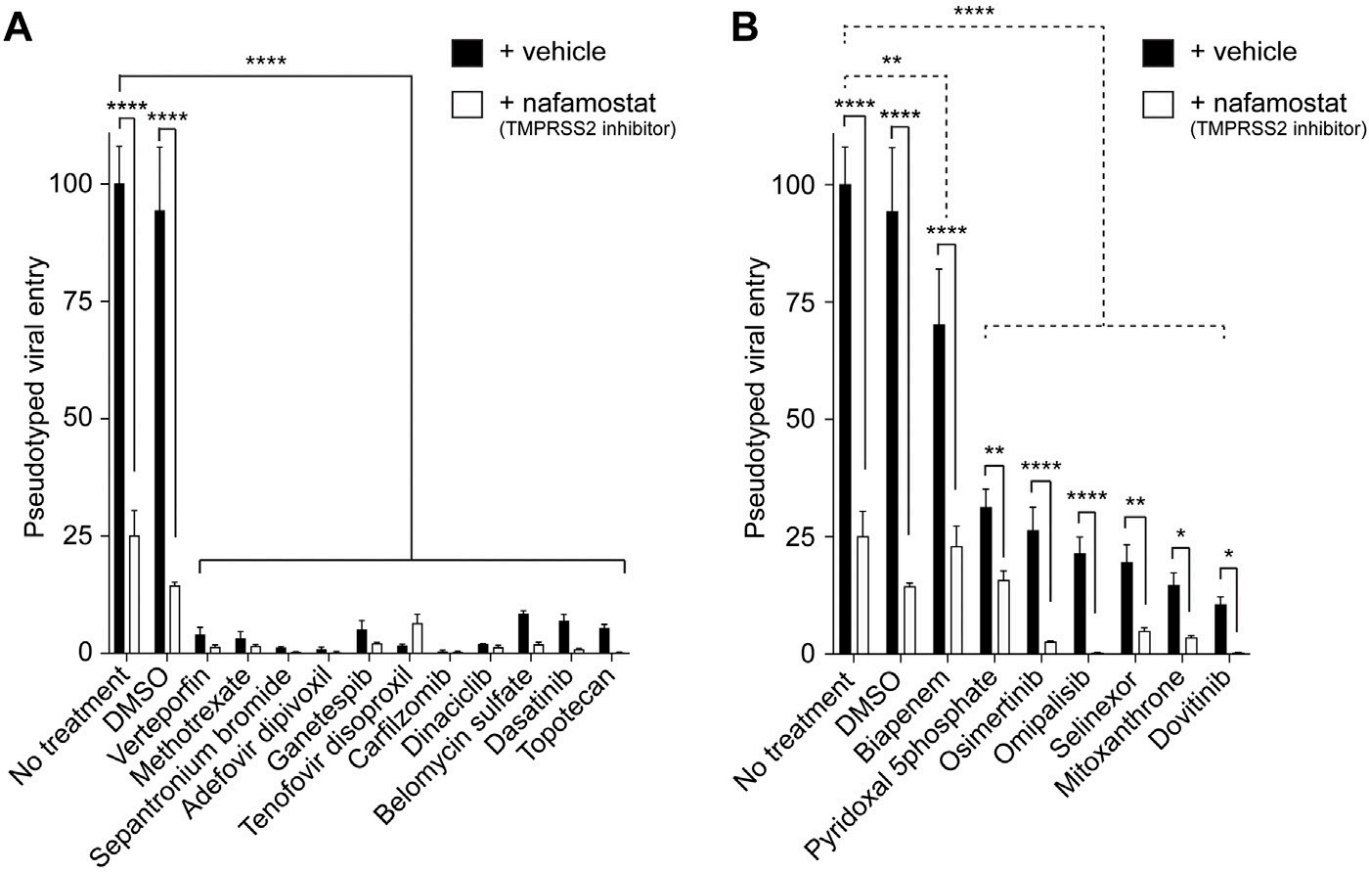
The effect of TMPRSS2 on the antiviral activities of the screen hits. **(A)** Graph showing the inhibitors of the pseudotyped SARS-CoV-2 virus entry into the cells unaffected by TMPRSS2 inhibition. The luciferase readings were normalized to their respective protein content. *n* = 3, two-way ANOVA test. Bonferroni’s multiple comparisons test, **** (*P* < 0.0001). Error bars = ±SEM. **(B)** Graph showing the inhibition of the entry of pseudotyped SARS-CoV-2 virus entry into the cells modulated by TMPRSS2 inhibition. The luciferase readings were normalized to their respective protein content. *n* = 3, two-way ANOVA test. Bonferroni’s multiple comparisons test comparing control vs. entry inhibitors (dotted line comparisons), ** (*P* = 0.0059), **** (*P* < 0.0001). Uncorrected Fisher’s LSD for ± nafamostat comparisons (solid line comparisons). **** (*P* < 0.0001, no treatment, DMSO, Biapenem, Osimertinib, and Omipalisib), ** (*P* = 0.0019, pyridoxal 5′-phosphate), ** (*P* = 0.0032, Selinexor), * (*P* = 0.0219, mitoxanthrone), * (*P* = 0.0335, Dovitinib) respectively. Error bars = ±SEM.

The pseudotyped viral entry experiments in the cells expressing both ACE2 and TMPRSS2 in the presence or absence of the screen hits with or without nafamostat revealed that our screen hits can be divided into two groups. The first group included 11 drugs, verteporfin, methotrexate, sepantronium bromide (YM-155), adefovir dipivoxil, ganetespib, tenofovir disoproxil, carfilzomib, dinaciclib, belomycin sulfate, dasatinib, and topotecan. The first group almost completely blocked the entry of the virus with no significant effect of TMPRSS2 inhibition (Figure 3A). These findings suggest that screen hits in the first group potently inhibit ACE2-and TMPRSS2-activity-dependent pseudotyped SARS-CoV-2 entry. The second group included seven drugs, biapenem, pyridoxal 5′-phosphate, osimertinib, omipalisib, selinexor, mitoxanthrone, and dovitinib. The second group partially blocked the entry of the virus with a significant synergistic effect of TMPRSS2 inhibition (Figure 3B). These findings suggest that screen hits in the second group potently inhibit ACE2-but not TMPRSS2-activity-dependent pseudotyped SARS-CoV-2 entry.

### Antiviral activity and cytotoxicity of biapenem, pyridoxal 5′-phosphate, adefovir dipivoxil, and dovitinib

We selected the screen hits that have not been previously reported for their ability to inhibit pseudotyped SARS-CoV-2 viral entry, namely, biapenem, pyridoxal 5′-phosphate, adefovir dipivoxil and dovitinib for further characterization.

**FIGURE 4:**
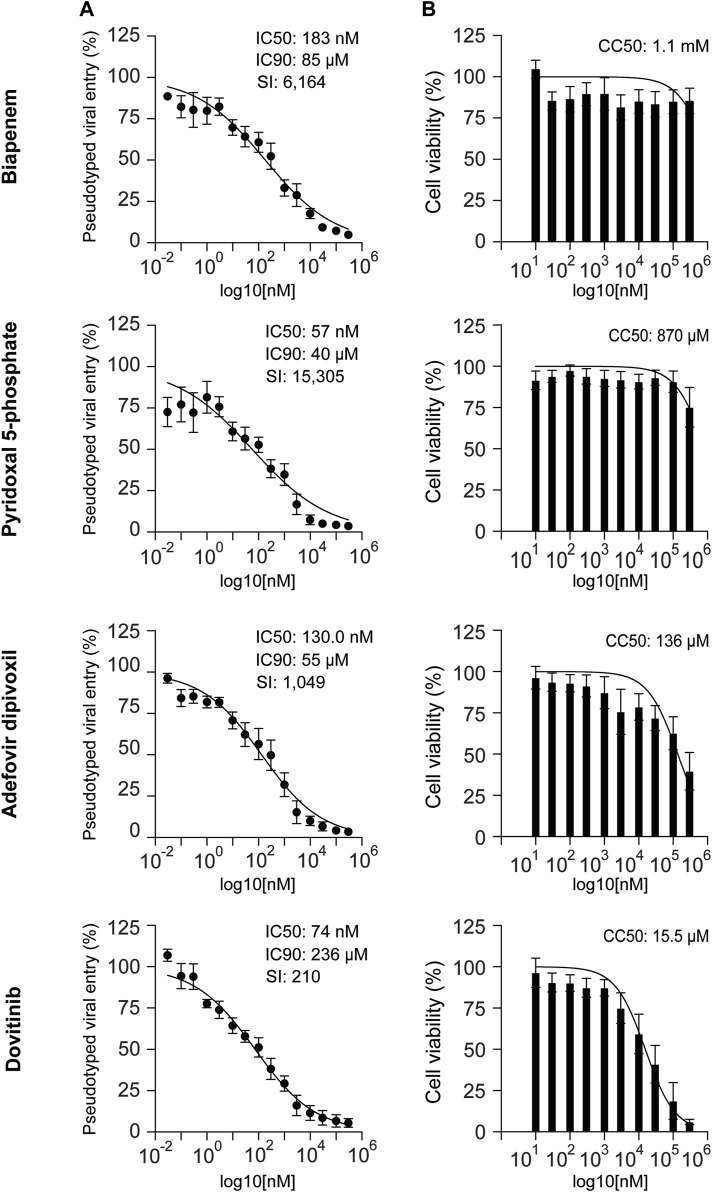
Antiviral activity and cytotoxicity of biapenem, pyridoxal 5′-phosphate, adefovir dipivoxil, and dovitinib. **(A)** Dose-response pseudovirus entry analysis of HEK-293-ACE2 cells treated with biapenem, pyridoxal 5′-phosphate, adefovir dipivoxil and dovitinib. The luciferase readings were normalized to the respective protein content. Three parameters non-linear regression analysis was done; *n* = at least 4. Error bars = ±SEM **(B)** Graphs showing the effect of biapenem, pyridoxal 5′-phosphate, adefovir dipivoxil and dovitinib on the cell-survival upon 48 h of treatment. Non-linear regression analysis was done: *n* = 8. Error bars = ±SEM. IC50: inhibitory concentration 50%. IC90: inhibitory concentration 90%. SI: selectivity index. CC50: concentration of cytotoxicity 50%.

To determine the potency of the selected drugs, we performed dose-response experiments using 15 drug concentrations ranging from 30pM to 300 μM in HEK-293 cells stably expressing hACE2. We found that the IC_50_ values of pyridoxil 5-phosphate, dovitinib, adefovir dipivoxil, and biapenem, are 57nM, 74 nM, 130 nM, and 183 nM, respectively. The results suggest that these drugs are potent inhibitors of ACE2-dependent pseudotyped SARS-CoV-2 viral entry into cells (Figure 4A).

We determined the cytotoxicity of these compounds in HEK-293 cells based on the quantification of ATP content as an index for metabolically active cells. Biapenem, pyridoxal 5′-phosphate, and adefovir dipivoxil did not show significant toxicity with CC_50_ values >100 μM. Dovitinib showed relatively more toxicity with CC_50_ value of 15.5 μM (Figure 4B).

Selectivity Index (SI) of an anti-viral drug is defined as the ratio between its cytotoxicity and anti-viral activity (CC_50_/IC_50_). It is considered that the biological efficacy of a drug is not due to its *in vitro* cytotoxicity when SI ≥10 (Vonthron-Senecheau et al., 2003; Muthaura et al., 2011). The optimal drug would exhibit cytotoxicity only at extremely high concentrations, while displaying antiviral activity at very low concentrations, resulting in a high selectivity index. This would allow it to eliminate the target virus at levels far below its cytotoxic threshold. The selectivity index is a commonly used parameter to indicate a compound’s *in vitro* antiviral effectiveness. We calculated the SI of the four selected drugs. We found that the SI of biapenem, pyridoxal 5′-phosphate, adefovir dipivoxil and dovitinib are 6,164, 15,305, 1,049, and 210, respectively.

## Discussion

The rapid spread of the SARS-CoV-2 virus and its ability to cause severe respiratory illness has highlighted the urgent need for effective treatments. Several effective vaccines have been developed and approved for use (Al-Fattah Yahaya et al., 2023; Wu et al., 2023; Corbett et al., 2020). However, certain patient groups with reduced immunity and the elderly do not get complete vaccine protection (Al-Fattah Yahaya et al., 2023; Wu et al., 2023). Moreover, as of 31 December 2023, only 67% of the world population was vaccinated with at least a complete primary series of a COVID-19 vaccine (World Health Organization). Hence, searching for effective therapies to treat COVID-19 patients remains ongoing.

Despite decades of modern biomedical research, the translation of bench discoveries into therapeutics is still lagging. The process of drug discovery and development average around 12 years with cost estimates range from $160 million to $4.5 billion per new drug (Schlander et al., 2021). The COVID-19 pandemic has expedited the development of vaccines and therapeutics to reduce the mortality and morbidity of this disease. Hence, this urgency caused drug developers to use drug repurposing as an alternative strategy (Ng et al., 2021). Drug repurposing seeks new uses outside the scope of the original medical indication for approved or investigational drugs. Recent FDA approval to repurpose the general antiviral remdesivir for treating COVID-19 is a successful example of drug repurposing (Govender and Chuturgoon, 2022). One approach to identify potential drugs for COVID-19 treatment is to screen FDA-approved drugs for their ability to inhibit viral entry. Viral entry inhibitors impede the initial step of viral infection, thus limiting viral replication and diminishing harm to the host.

In this study, we performed a drug repurposing screen of ∼2,500 FDA-approved compounds targeting ACE2-dependent pseudotyped SARS-CoV-2 entry into cells. We utilized a previously established system to screen the compound library at 10 μM against an MLV virus pseudotyped with the SARS-CoV-2S encoding a luciferase reporter in HEK-293-ACE2 cells (Mediouni et al., 2022). The hits from the first screen were confirmed using the same system at 1 μM. We identified 18 FDA-approved hits, four of which were not previously reported to inhibit SARS-CoV-2 entry. We tested the 18 hits in HEK-293-ACE2-TMPRSS2 cells with and without the TMPRSS2 inhibitor nafamostat. We found that 11 hits potently block pseudotyped SARS-CoV-2 entry in ACE2-TMPRSS2 expressing cells (Figure 3A). The other seven hits reduce pseudotyped SARS-CoV-2 entry in ACE2-TMPRSS2 expressing cells, showing synergistic inhibition of viral entry when combined with nafamostat (Figure 3B). The differential inhibition effect of the drugs on pseudotyped SARS CoV-2 entry may be attributed to their specific mechanisms of action.

Drugs inhibiting ACE2-and TMPRSS2- dependent entry (Figure 3A) might act through multi-targets. They might block the ACE2 spike protein interaction and obstruct TMPRSS2-mediated spike protein priming, disrupting the membrane fusion. Alternatively, inactivation of the viral particles or a post-entry step might be the target explaining the insensitivity to TMPRSS2 inhibition. For example, Topotecan inhibits SARS-CoV-2 pseudovirus entry *in vitro* and *in vivo* by decreasing the protein levels of ACE2 and TMPRSS2 (Tong et al., 2023). Verteporfin effectively inhibited the SARS-CoV-2 entry *in vitro* and prevented SARS-CoV-2 infection in a humanized ACE2 mouse model. Verteporfin molecule has a porphyrin ring structure that binds to the ACE2 receptor on the host cell, interfering with its binding with the viral S protein (Gu et al., 2021). Other studies showed that verteporfin inhibits SARS-CoV-2 replication, a post-entry step (Garcia et al., 2022; Wang et al., 2024). Methotrexate has also been reported to inhibit SARS-CoV-2 entry *in vitro* and suppress lung infection and inflammation in a Syrian hamster model for COVID-19. Methotrexate has a multi-target action, including inhibiting SARS-CoV-2 entry, replication, and lung inflammation (Chen et al., 2022). YM-155 was found to have potent anti SARS-CoV-2 activity by inhibiting the viral papain-like protease (PLpro). PLpro processes the viral replicase polyprotein into functional units (Zhao et al., 2021). Adefovir and tenofovir disoproxil fumarate are known as nucleotide analog reverse transcriptase inhibitors (Chen et al., 2020; Park et al., 2020). In another study conducted on human lungs alveolar epithelial cells and validated on hamster model for SARS CoV-2, the HSP90 inhibitor, ganetespib inhibited SARS-CoV-2 viral replication *in vitro* and reduced tissue pathology *in vivo* (Teixeira Alves et al., 2024). Carfilzomib is known as an effective proteasome inhibitor. The main protease of SARS-CoV-2, known as 3CL^pro^, is essential for its replication. Through virtual screening technology and molecular dynamics simulations, carfilzomib demonstrated strong binding affinity to the active sites of 3CL^pro^, suggesting its potential to inhibit 3CL^pro^ activity (Wang Q. et al., 2022). A strong anti-viral activity for the CDK inhibitor dinaciclib was found in 2 cell lines, Vero E6 and A549-ACE2. As per the study, dinaciclib shows its anti SARS CoV-2 effect by inhibiting CDK9, thereby disrupting viral transcription, and replication. This process potentially reduces the overall SARS-CoV-2 infection (Bouhaddou et al., 2020). Dasatinib was identified as ADP-ribosylhydrolase inhibitor for SARS-CoV-2 and MERS-CoV. (Dasovich et al., 2022). In another study, a combination of dasatinib and quercetin was shown to reduce SARS-CoV-2 related mortality in murine K18-ACE2 model. Dasatinib/quercetin markedly elevated the number of mice exhibiting reduced levels of pro-inflammatory cytokines linked to COVID-19-related mortality (Pastor-Fernandez et al., 2023).

On the other hand, drugs that inhibit only ACE2-dependent entry (Figure 3B) might block the interaction between the spike protein and ACE2 or inhibit the internalization of the ACE2-viral particle complex without affecting TMPRSS2 function (Jackson et al., 2022; Wang L. et al., 2022; Baby et al., 2021). For example, the nuclear export protein (XPO1) inhibitor, selinexor diminished SARS-CoV-2 entry *in vitro* and reduced viral load *in vivo* likely through inhibiting XPO1, leading to nuclear localization of ACE2 (Kashyap et al., 2021). Hence, selinexor inhibits ACE2-dependent SARS-CoV-2 entry by relocating ACE2 from the surface membrane to the nucleus. Mitoxantrone was found to inhibit SARS-CoV-2 infection by modulating a heparan sulfate-spike complex in hACE-HEK293T cells. By modulating this spike-heparan sulfate complex, it reduces the ability of SARS CoV-2 to bind to the host cells, thereby reducing viral entry (Zhang et al., 2022). Interestingly, heparan sulfate facilitates SARS-CoV-2 spike protein binding to ACE2 enhancing viral entry (Clausen et al., 2020; Kalra and Kandimalla, 2021; Zhang et al., 2023). Osimertinib has been reported effective as a broad-spectrum inhibitor for spike-mediated entry of pseudotyped SARS-CoV-2 and MERS-S particles (Chen et al., 2020). It is known to be a tyrosine kinase inhibitor of the T790M mutant of epidermal growth factor receptor (EGFR), which is activated along with its major downstream signaling pathway, the mitogen-activated signaling pathway (MAPK) during SARS-CoV-2 infection (Engler et al., 2023; Palakkott et al., 2023). Interestingly, SARS-CoV-2 spike protein receptor binding doman (RBD) induces EGFR-ACE2 cross talk, suggesting that EGFR and MAPK signaling are related to SARS-CoV-2 entry. Also, omipalisib showed potent inhibition of pseudotyped SARS-CoV-2 virus entry in Vero E6 cells but the exact mechanism remains unclear (Patten et al., 2022; Jang et al., 2021).

Our dose-response inhibition of ACE2-dependent pseudotyped viral entry and toxicity assays provided additional insights into the potential therapeutic application of the four newly identified anti-SARS-CoV-2 candidates: Biapenem, pyridoxal 5′-phosphate, Adeovir dipivoxil and Dovitinib. Biapenem pyridoxal 5′-phosphate, and Adefovir dipivoxil exhibited low toxicity and potent inhibitory effects on viral entry. Dovitinib also showed potent inhibition of viral entry but had relatively higher toxicity profile, suggesting a need for careful dosage optimization in potential therapeutic applications. These four selected FDA-approved drugs were primarily in use to treat different conditions. In the forthcoming paragraphs, we have discussed their primary usages and mechanism of action.

Biapenem is a carbapenem broad-spectrum antibiotic that inhibits bacterial cell wall synthesis. Biapenem is useful for the treatment of severe infections such as sepsis, lower respiratory infections, tuberculosis, urinary tract infections, intra-abdominal and genitourinary infections (Chen et al., 2024; Vora et al., 2022). A recent drug repurposing study found biapenem to inhibit particulate matter-induced lung injury. In this context, biapenem suppresses autophagy by inducing PI3K/Akt/mTOR pathway. Also, biapenem reduces particulate matter induced TLR4 and MyD88 upregulation, which correlates with reduction of inflammation (Lee et al., 2020). Moreover, biapenem might act as a potent adjunct innate and adaptive immunomodulator (Pahuja et al., 2023). Here, we found that biapenem potently inhibits ACE-2-dependent pseudotyped SARS-CoV-2 entry into cells with an IC_50_ of 183 nM and IC_90_ of 80.87μM, with a CC_50_ of 1.1 mM. The SI of biapenem as calculated is 6,164, making it a potential candidate for further investigation in COVID-19 treatments. Biapenem dose as an antibiotic is 300 mg twice a day (Takata et al., 2004). To date, there are no reports of antiviral activity of biapenem. Based on our experiments, we hypothesize that biapenem anti-SARS-CoV-2 activity is mediated by inhibiting ACE2-dependent viral entry. Hence, biapenem might interfere with the binding of SARS CoV-2 spike protein with ACE2 or inhibit ACE2-SARS-CoV-2 complex internalization in the cell.

Pyridoxal 5′-phosphate, the active form of vitamin B6, has shown potential as an antiviral agent, particularly against HIV-1 by binding to the CD4 protein and inhibiting the gp120-CD4 interaction (Salhany and Schopfer, 1993). Pyridoxal 5′-phosphate antiviral efficacy is further supported by its ability to modify HIV-1 integrase, impairing viral replication (Guo et al., 1994; Williams et al., 2005). Furthermore, several studies demonstrate anti-inflammatory activities for pyridoxal 5′-phosphate (Katunuma et al., 2000; Sakakeeny et al., 2012; Zhang et al., 2016; Qian et al., 2017; Shan et al., 2020; Du et al., 2020; Zhu et al., 2023; Turnquist, 2023). Hyper-inflammation triggers severe COVID-19 and high mortality from the disease (Vollbracht and Kraft, 2022; Stroz et al., 2024). Our results show potent inhibition of pseudotyped SARS-CoV-2 viral entry into cells using pyridoxal 5′-phosphate with an IC_50_ of 57 nM, IC_90_ of 40 μM and a CC_50_ of 870 μM. The average dietary supplement dose recommended for vitamin B6 intake is about 1.5 mg/day in women and 2 mg/day in men (NIH factsheet). Therapeutically, it is recommended at a dose of 30 mg/kg/day enterally, divided into 3-4 doses in epileptic neonatal patients (Amanda, 2019). Given its SI of 15,305, potent antiviral and anti-inflammatory activities, Pyridoxal 5′-phosphate seems to be a promising candidate for combination therapy in COVID-19. Like biapenem, we hypothesize that pyridoxal 5′-phosphate anti-SARS-CoV-2 activity is mediated by inhibiting ACE2-dependent viral entry.

Adefovir dipivoxil, a nucleotide analogue with demonstrated antiviral efficacy against HBV, HIV, and poxviruses, effectively reduces viral DNA levels in various models and is well-tolerated in clinical settings (Julander et al., 2002; Cullen et al., 2001; Dsouza et al., 2023). The IC_50_, IC_90_, and CC_50_ for adefovir dipivoxil for SARS-CoV-2 are 130 nM, 55 μM,and 136 μM, respectively. These values, along with the SI of 1,049, suggest that it might be a good candidate for combination therapy in COVID-19 management. Adefovir dipivoxil is shown to be effective at a dose of 10 mg/day in adults to treat HIV-1 and HPV-B (Guo et al., 1994; Wu et al., 2008). In coronaviruses like SARS-CoV, MERS-CoV, and SARS-CoV-2, RNA-dependent RNA polymerase (RdRp) is highly conserved. RdRp is crucial for replicating and transcribing a viral RNA genome. Hence, it can be a potential target to inhibit coronavirus infection. In a study reported by Min et al., adefovir dipivoxil has shown significant efficacy in inhibiting SARS-CoV-2 entry into the cells by RdRp inhibition, suggesting a plausible mechanism of action (Min et al., 2021). Alternatively, adefovir dipivoxil might be showing its effect by hydrolyzing into adefovir diphosphate moiety which inhibits deoxyadenosine triphosphate as a substrate for reverse transcriptase, causing a chain termination of the growing DNA chain; hence, impeding the viral replication (Noble and Goa, 1999).

Dovitinib (TKI258), an investigational oral tyrosine kinase inhibitor targeting multiple receptors including FGF, VEGF, and PDGF receptors, shows strong inhibitory activity against these targets with *in vitro* IC_50_ values of approximately 10 nM (Motzer et al., 2014; Chamberlin et al., 2016). It has shown potential in treating various cancers by inhibiting these pathways, which are often implicated in tumor growth and resistance to other therapies. In our study, it has shown significant inhibitory effects against SARS-CoV-2 with an IC_50_ value of 74 nM, IC_90_ of 236 μM and a CC_50_ of 15.5 μM. Considering the SI value of dovitinib to be 210, it could be cautiously considered as a candidate for combination therapy against COVID-19. According to a phase I/II and pharmacodynamic study of dovitinib, a dose of 400 mg/d was shown to be safe and clinically effective (Kim et al., 2011). In another study, treatment of patients with squamous cell cancer (SCC) of the lung with dovitinib (500 mg per day) was shown to be modestly effecttive (Lim et al., 2016). Dovitinib has been reported to show anti-cancerous activity through IFN-γ signaling (Cao et al., 2023). However, there are no reports to date showing its effect as an antiviral drug. Based on our study, we hypothesize that dovitinib anti-SARS-CoV-2 activity is mediated by inhibiting ACE2-dependent viral entry.

In summary, we have identified 18 FDA-approved drugs which effectively inhibit the ACE2-dependant entry of pseudotyped SARS-CoV-2 into the cells. 14 of these drugs have recently been reported. Our screen confirms their role as anti-SARS-CoV-2 candidates. We identified four novel anti-SARS-CoV-2 candidates. Lessons from the HIV and hepatitis C show that combined antiviral therapy allows higher antiviral efficacy than single drugs (Hollande et al., 2020; Margolis and Hazuda, 2013). Pyridoxal 5′-phosphate, Dovitinib, Adeovir dipivoxil, and Biapenem might help develop combined anti-SARS-CoV-2 therapies.

## Data Availability

The original contributions presented in the study are included in the article/supplementary material, further inquiries can be directed to the corresponding author.
